# Prognostic Significance and Therapeutic Potential of SERPINE1 in Head and Neck Squamous Cell Carcinoma

**DOI:** 10.1002/cam4.70605

**Published:** 2025-01-16

**Authors:** Changyu Zhu, Heshu Liu, Zhixin Li, Yijun Shi, Jingyang Zhao, Yuping Bai, Qian Chen, Wei Li

**Affiliations:** ^1^ Cancer Center Beijing Tongren Hospital, Capital Medical University Beijing China; ^2^ Department of Pathology Beijing Tongren Hospital, Capital Medical University, Beijing Key Laboratory of Head and Neck Molecular Diagnostic Pathology Beijing China; ^3^ Thorgene co. Ltd. Beijing China

**Keywords:** HNSCC, immune, prognosis, SERPINE1

## Abstract

**Background:**

This study aims to elucidate the expression pattern of SERPINE1, assess its prognostic significance, and explore potential therapeutic drugs targeting this molecule.

**Methods and Results:**

In this study, we delved into the variations in gene mutation, methylation patterns, and expression levels of SERPINE1 in head and neck squamous cell carcinoma (HNSCC) and normal tissues, leveraging comprehensive analyses of The Cancer Genome Atlas (TCGA) and Gene Expression Omnibus (GEO) datasets. The connection between the biological function of the gene and prognosis was scrutinized through immune infiltration and enrichment analyses. Concurrently, we assessed the potential therapeutic value of SERPINE1 through drug sensitivity analysis. It was observed that, particularly in human papillomavirus (HPV) negative HNSCC, SERPINE1 exhibited elevated expression levels, correlating with poorer prognosis. The infiltration levels of eight cell types, such as eosinophils, Tgd, and macrophages, showed a positive correlation with SERPINE1 expression, whereas infiltration levels of four cell types, including cytotoxic cells, B cells, and pDCs, displayed a negative correlation. Furthermore, copy number variations of SERPINE1 were primarily characterized by homologous amplification, positively correlating with its expression, while methylation showed an inverse correlation. The outcomes of drug sensitivity analysis underscored the potential of SERPINE1 as a therapeutic target.

**Conclusion:**

Elevated expression of SERPINE1 in HNSCC is intricately linked with adverse prognostic outcomes and has the potential to influence the immune microenvironment. Subsequent investigations are imperative to fully elucidate the prognostic implications of SERPINE1 as a biomarker and to unlock its therapeutic promise as a target for intervention.

## 1 Introduction

Head and neck squamous cell carcinoma (HNSCC) ranks as the sixth most common cancer worldwide, with an annual incidence of over 680,000 new cases [1]. The 5‐year survival rate hovers around 50%, with more than 90% of cases originating from the epithelium of the oral cavity, oropharynx, larynx, and hypopharynx [2, 3]. Prominent risk factors include tobacco use, alcohol consumption, and HPV infection [4]. Despite advances in treatment modalities, overall survival rates (OS) for HNSCC remain suboptimal [5]. The exploration of novel biomarkers and molecular targets holds promise for improving therapeutic outcomes.

The serine protease inhibitor SERPINE1 acts by inhibiting tissue plasminogen activator (tPA) and urokinase‐plasminogen activator (uPA) [6]. Extensive research has illuminated the role of SERPINE1 in cancer progression and metastasis, highlighting its angiogenic potential, facilitation of growth and migration, and suppression of apoptosis, ultimately fostering tumor growth, cancer cell survival, and metastatic dissemination [7]. Notably, SERPINE1 emerges as a dependable prognostic biomarker across various cancer types, including colon [8], breast [9], pancreatic [10], bladder [11], non‐small cell lung [12], and low‐grade gliomas [13]. In colon cancer, SERPINE1 contributes to microenvironmental remodeling and immune cell infiltration [14], while heightened levels drive tumor cell invasion and proliferation via the induction of epithelial‐mesenchymal transition (EMT) in gastric cancer [15]. Furthermore, a few studies have suggested a potential correlation between SERPINE1 and the progression of gastric cancer owing to its pro‐angiogenic effects [16]. Earlier investigations also hint at a relationship between SERPINE1 and the prognosis of HNSCC [17].

Although the SERPINE1 has been extensively studied in other cancers, there is limited evidence of its role in HNSCC. The expression, function, and potential mechanisms of action of SERPINE1 in HNSCC and normal tissues remain unclear. Therefore, the aim of this study is to investigate the expression and function of SERPINE1 in HNSCC, and to ascertain its potential utility as a biomarker and therapeutic target.

## 2 Materials and Methods

### 2.1 Target Gene Screening

This study obtained TPM data for HNSCC from TCGA and conducted separate analyses, including 504 cancer samples and 44 adjacent normal samples. Additionally, 43 cancer samples were compared to their adjacent normal samples to validate SERPINE1 expression. Prognostic assessment using the “limma” R package focused on overall survival (OS) and disease‐specific survival (DSS) correlations. Validation was performed using GSE41613 dataset for OS and DSS, and GSE65858 dataset for OS and DFS correlations from Gene Expression Omnibus (GEO).

### 2.2 The Association Between SERPINE1 and Clinical Features

SERPINE1 expression with clinical indicators in both TCGA‐HNSCC and GSE65858 datasets were compared to the consistency and reliability across datasets were evaluated.

### 2.3 Functional and Pathway Enrichment Analysis of SERPINE1

To understand SERPINE1's role in tumor and normal tissues, we analyzed the HNSCC dataset from TCGA. Differential gene expression analysis compared gene levels between cancerous and normal tissues, identifying differentially expressed genes (*p* < 0.05, |logFC| > 2). Using the “ClusterProfiler” package, we conducted Gene Ontology (GO) and Kyoto Encyclopedia of Genes and Genomes (KEGG) pathway enrichment analyses [18]. Results were visualized with “ggplot2”. Additionally, Gene Set Enrichment Analysis (GSEA) was performed using the same dataset, evaluating SERPINE1 enrichment with FDR *q* < 0.25 and *p* < 0.05.

### 2.4 Protein Interaction Network Analysis

To identify the SERPINE1 gene within a network, we utilized the Protein–Protein Interaction (PPI) network from the STRING database (https://string‐db.org) to analyze SERPINE1 and its closely related genes, visualizing their interactions. The analysis was conducted using a high confidence threshold (0.700) for the interaction score.

### 2.5 Immune‐Infiltration Analysis

To assess immune infiltration, we employed the R‐GSVA immune infiltration algorithm alongside the Spearman statistical method, utilizing immune cell markers referenced in the Immunity article [19]. The matrix and immune scores were computed using the R package “estimate” (version 1.0.13).

### 2.6 Genetic and Epigenetic Characterization of SERPINE1: SNV, CNV and Methylation Analysis

The GSCA database utilized TCGA's HNSCC data to analyze the Single Nucleotide Variations (SNV), Copy Number Variations (CNV), and methylation patterns of SERPINE1. SNV analysis encompassed seven mutation types: Missense_Mutation, Nonsense_Mutation, Frame_Shift_Ins, Splice_Site, Frame_Shift_Del, In_Frame_Del, and In_Frame_Ins. CNV analysis was conducted using GISTIC2.0 on sample data to identify amplification or deletion regions exhibiting significant changes between patient groups. Methylation data for SERPINE1 was obtained from the TCGA database as Illumina Human Methylation 450k level 3 data. Prior to differential methylation analysis, correlation analysis was performed to filter out sites negatively correlated with gene expression. The *p*‐values were estimated using t‐tests and further adjusted using FDR.

### 2.7 Assessment of Drug Sensitivity

The GSCA database facilitated drug sensitivity analysis, exploring how genomic aberrations influence clinical responses to treatment and serve as potential biomarkers for drug screening. This study integrated gene expression and drug sensitivity data from GDSC (Genomics of Drug Sensitivity in Cancer) and CTRP (Cancer Therapeutics Response Portal). Gene expression within the dataset was correlated with small molecule/drug sensitivity (IC50) using Spearman correlation analysis [20].

### 2.8 Immunohistochemistry and Clinical Indicators Associated With SERPINE1

We analyzed the correlation between SERPINE1 scores and clinical indicators by combining immunohistochemistry scores. Tissue microarrays comprised 53 primary HNSCC tumors and 9 para‐cancerous tissues sourced from Shanghai Outdo Biotechnology. Tissue sections underwent a 15‐min xylene dewaxing and alcohol hydration process (100%, 100%, 80%, 75%). Antigen retrieval was performed using citrate solution (B0034, China), followed by a 30‐min blocking step with goat serum. Sections were then incubated overnight at 4°C with a primary antibody against SERPINE1 (1:300 dilution, 66.261‐1‐Ig, Proteintech). Subsequently, sections were incubated for 50 min with the corresponding secondary antibody (HRP‐labeled, anti‐rabbit/anti‐mouse, ab205718/ab205719, Abcam) at room temperature. Immunohistochemistry (IHC) signals were visualized using the 3,3′‐diaminobenzidine (DAB) chromogenic kit (K3468, DAKO), with IHC results interpreted based on hematoxylin‐stained blue nuclei and brownish‐yellow DAB‐positive expression.

Two pathologists independently evaluated the immunohistochemical staining results. The SERPINE1 expression score was determined by averaging their assessments, taking into account the percentage of positively stained tumor cells and staining intensity. Positive cell density was assessed under a low‐power microscope, with 0 points assigned for no positive tumor cells, 1 point for ≤ 35%, 2 points for 35%–70%, and 3 points for > 70%. Staining intensity categories were defined as 0 (no stain), 1 (weak: light yellow), 2 (moderate: yellow‐brown), and 3 (strong: brown). The staining score was calculated by multiplying the staining intensity by the proportion of positive tumor cells.

### 2.9 Statistical Analysis

In this study, statistical analyses were performed using R (version 4.2.1) and AI (Adobe Illustrator) drawing software. The statistical tests utilized included the Wilcoxon rank‐sum test and Mann–Whitney *U*‐test (two‐sided). For specific analyses, Pearson's correlation coefficient or Fisher's exact test (two‐sided) was employed. A significance level of *p* < 0.05 was used to determine statistical significance.

## 3 Results

### 3.1 Upregulated Expression of SERPINE1 in HNSCC Associated With Poor Prognosis

**FIGURE 1:**
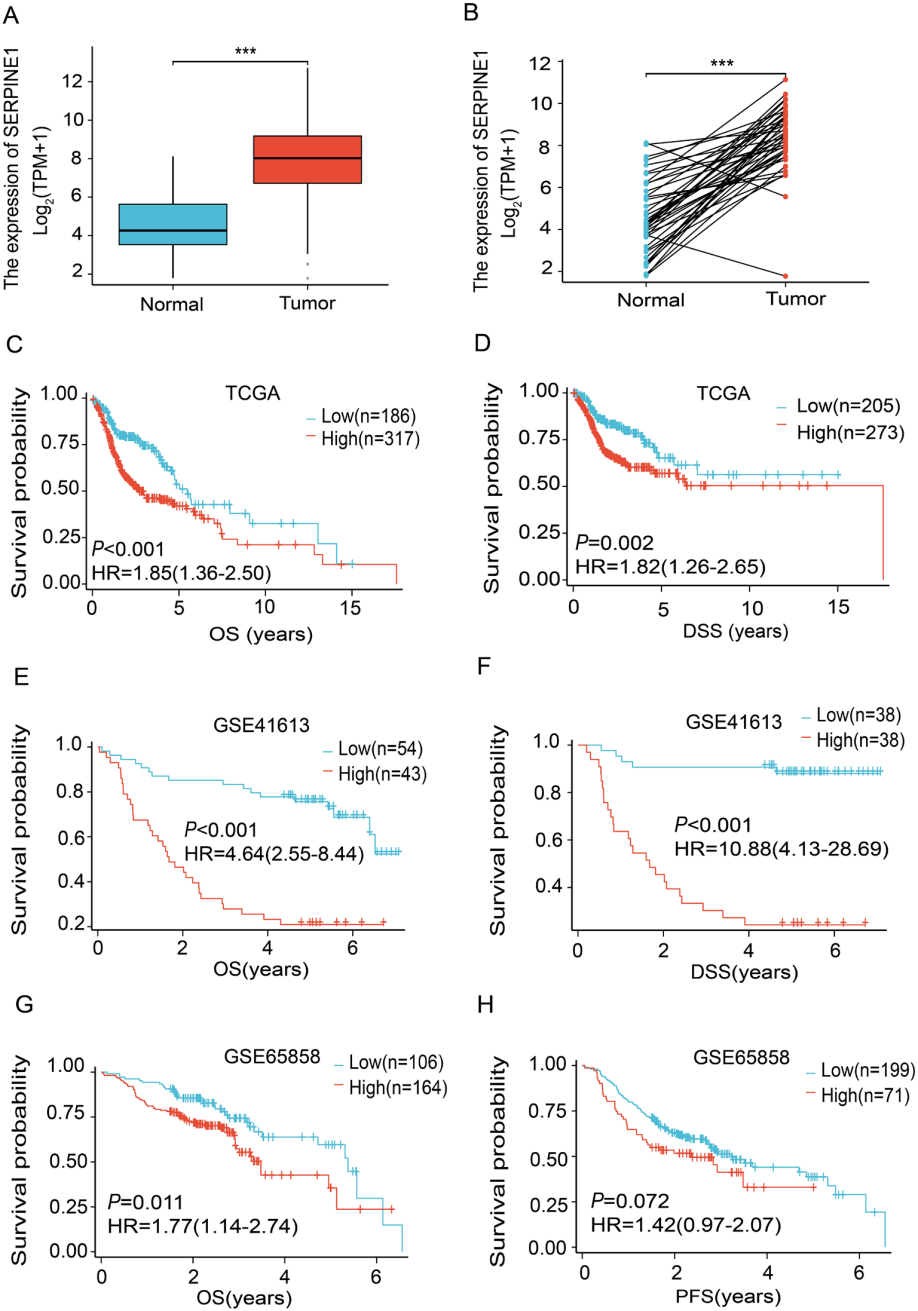
The relationship between SERPINE1 expression and prognosis. (A) SERPINE1 gene expression in HNSCC and normal tissues (TCGA); (B) SERPINE1 gene expression in HNSCC and adjacent cancer tissues (TCGA); (C) Impact of SERPINE1 gene expression on OS (TCGA); (D) impact of SERPINE1 gene expression on DSS (TCGA); (E) impact of SERPINE1 gene expression on OS (GSE41613); (F) Impact of SERPINE1 gene expression on DSS (GSE41613); (G) Impact of SERPINE1 gene expression on OS (GSE65858); (H) Impact of SERPINE1 gene expression on PFS (GSE65858). * *p* < 0.05, ** *p* < 0.01 and *** *p* < 0.001.

The expression of SERPINE1 in HNSCC was significantly increased compared with that in normal tissues, as well as in the comparison between HNSCC and paired adjacent cancer tissues (Figure 1A,B). Clinical data analysis unveiled a substantial negative correlation between SERPINE1 expression and OS and DSS in HNSCC (Figure 1C,D). Similarly, analysis of the GSE41613 dataset revealed a significant negative correlation between SERPINE1 expression and OS and DSS (Figure 1E,F). In the GSE65858 dataset, SERPINE1 expression was found to be correlated with OS in HNSCC (*p* < 0.05), while no significant association was observed with Progression‐Free Survival (PFS) (Figure 1G,H).

### 3.2 The Association Between SERPINE1 and Clinical Features

**FIGURE 2:**
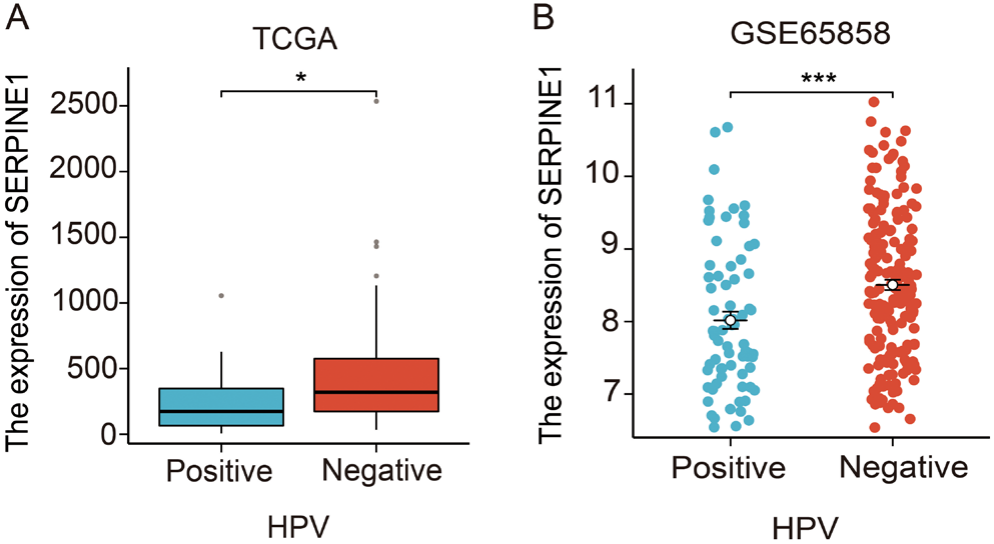
The association between SERPINE1 and clinical features. (A, B) The relationship between HPV status and SERPINE1 expression (TCGA and GSE65858 data) *p < 0.05, **p < 0.01, ***p < 0.001.

We utilized the clinical data from TCGA‐HNSCC and GSE65858 (Table 1), and analysis of TCGA‐HNSCC and GSE65858 data revealed that the expression of SERPINE1 showed no significant correlation with patient gender, age, T stage, N stage, or pathological stage (Figure S1A–F). However, the HPV status of patients was found to be correlated with SERPINE1 expression. In both datasets, SERPINE1 was notably upregulated in HPV‐negative patients (*p* < 0.05) (Figure 2A,B).

**TABLE 1:** The clinical data from TCGA‐HNSCC and GSE65858.

	TCGA (*n* = 504)	GSE65858 (*n* = 270)
Gender, *n* (%)		
Female	134 (26.6%)	47 (17.4%)
Male	370 (73.4%)	223 (82.6%)
Age, *n* (%)		
≤ 60	247 (49.0%)	158 (58.5%)
> 60	256 (50.8%)	112 (41.5%)
Unknown	1 (0.2%)	
Histologic grade, *n* (%)		
G1 and G2	363 (72.0%)	363 (72.0%)
G3 and G4	121 (24.0%)	121 (24.0%)
Unknown	20 (4.0%)	20 (4.0%)
Alcohol history, *n* (%)		
No	159 (31.5%)	31 (11.5%)
Yes	334 (66.3%)	239 (88.5%)
Unknown	11 (2.2%)	
Smoker, *n* (%)		
No	113 (22.4%)	48 (17.8%)
Yes	381 (75.6%)	222 (82.2%)
Unknown	10 (2.0%)	
Pathologic T stage, *n* (%)		
T1	45 (8.9%)	35 (13.0%)
T2	135 (26.8%)	80 (29.6%)
T3	96 (19.0%)	58 (21.5%)
T4	172 (34.1%)	97 (35.9%)
Unknown	56 (11.1%)	
Pathologic N stage, *n* (%)		
N0	171 (33.9%)	94 (34.8%)
N1	66 (13.1%)	32 (11.9%)
N2	167 (33.1%)	132 (48.9%)
N3	7 (1.4%)	12 (4.4%)
Unknown	93 (18.5%)	
Pathologic stage, *n* (%)		
Stage I and Stage II	95 (18.8%)	55 (20.4%)
Stage III and Stage IV	341 (67.7%)	215 (79.6%)
Unknown	68 (13.5%)	
HPV, *n* (%)		
Positive	19 (3.8%)	73 (27.0%)
Negative	64 (12.7%)	196 (72.6%)
Unknown	421 (83.5%)	1 (0.4%)
Lymphovascular invasion, *n* (%)		
No	220 (43.7%)	
Yes	122 (24.2%)	
Unknown	162 (32.1%)	

### 3.3 Analysis of Differentially Expressed Genes Associated With SERPINE1 and Their Functional and Pathway Enrichment

**FIGURE 3:**
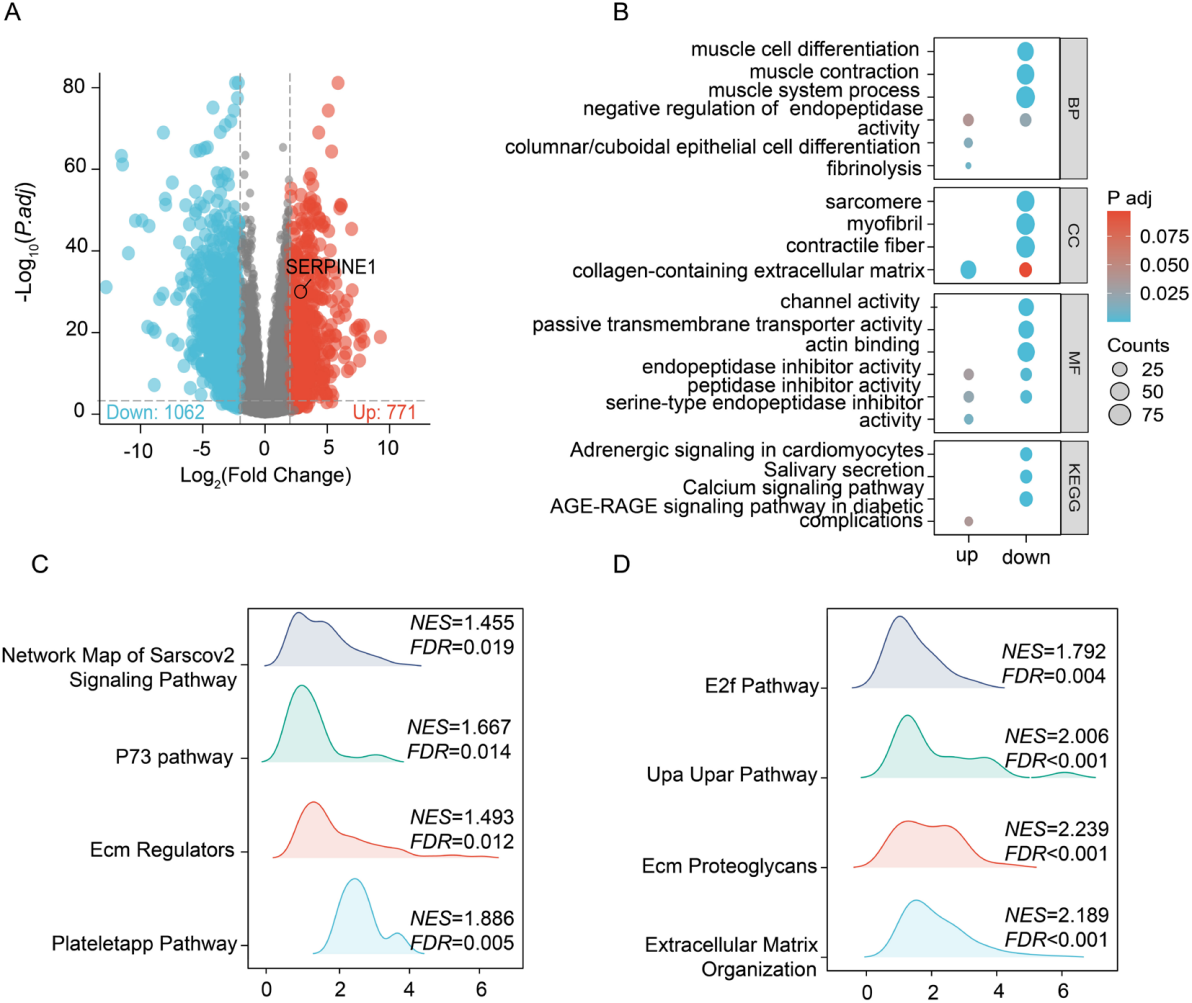
Visualization of Enriched Pathways. (A) Volcano plot illustrating the differential expression of genes between HNSCC and normal tissues; (B) GO and KEGG analyses highlighting SERPINE1 and genes with low expression; (C, D) GSEA showing enrichment analysis of SERPINE1.

Differential gene expression was assessed by categorizing gene expression levels between cancer and normal tissues. Among them, 1062 genes were significantly down‐regulated and 771 genes were significantly up‐regulated (*p*.adjust < 0.05, |logFC| > 2), while SERPINE1 was involved in the up‐regulated pathway (Figure 3A). GO and KEGG analyses revealed biological functions (BF) related to SERPINE1, including fibrinolysis, columnar/cuboidal epithelial cell differentiation, and negative regulation of endopeptidase activity. In terms of cellular component (CC), SERPINE1 was associated with the collagen‐containing extracellular matrix, while its molecular function (MF) included serine‐type endopeptidase inhibitor activity, peptidase inhibitor activity, and endopeptidase inhibitor activity. KEGG analysis indicated that the upregulated genes were significantly enriched in the AGE‐RAGE signaling pathway in diabetic complications pathway (Figure 3B). GSEA demonstrated enrichment of the SERPINE1 gene based on criteria of false discovery rate (FDR, *q*‐value) < 0.25 and *p*.adjust < 0.05. Enriched pathways included Extracellular Matrix Organization, ECM Proteoglycans, UpaUpar Pathway, E2f Pathway, Plateletapp Pathway, ECM Regulators, P73 Pathway, and Network Map of SARS‐CoV‐2 Signaling Pathway (Figure 3C,D).

### 3.4 Key Genes Interacting With SERPINE1

**FIGURE 4:**
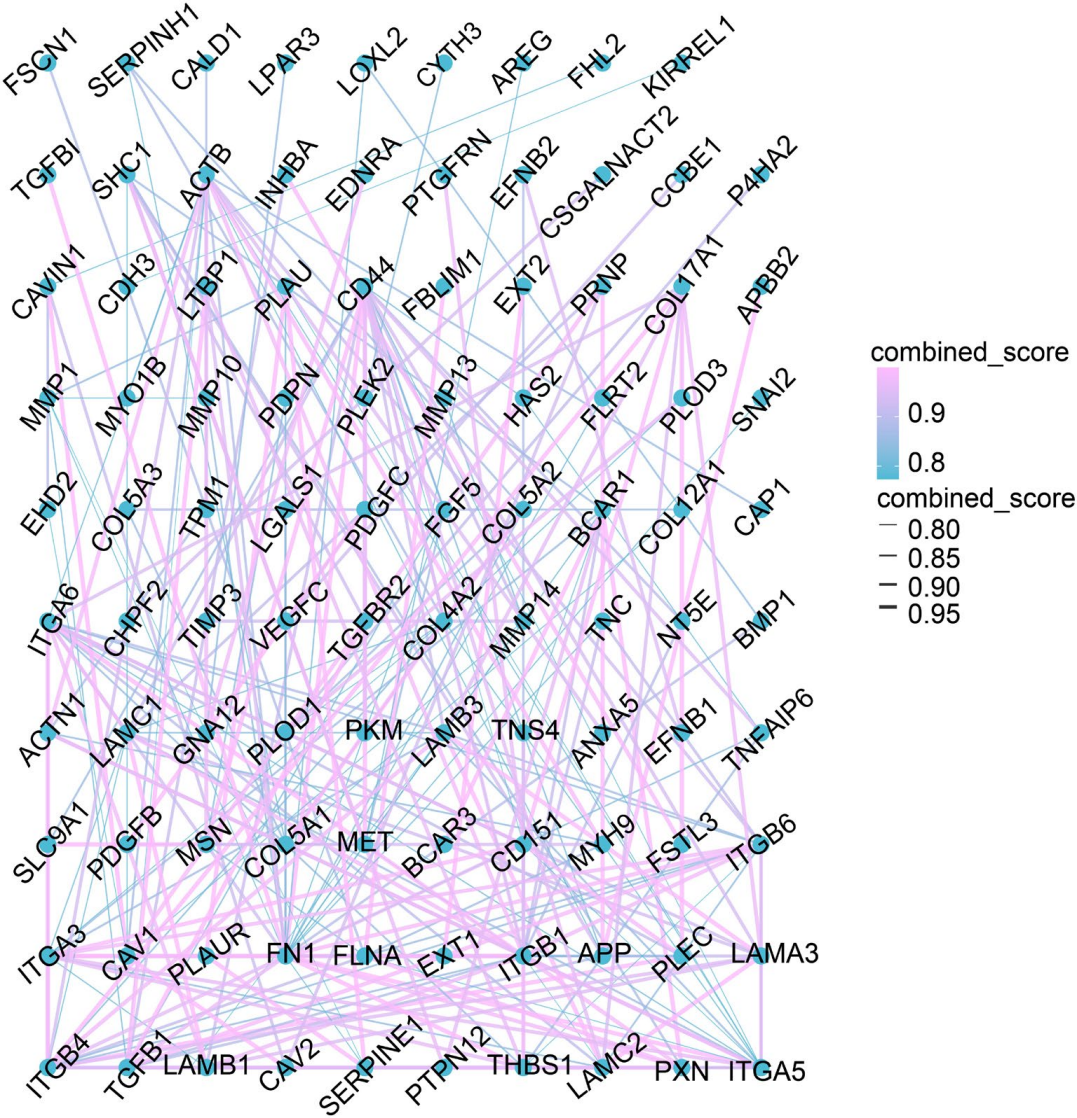
Protein–protein interaction between SERPINE1 and related genes.

By visualizing the PPI network of the selected genes, it can be seen that SERPINE1 mainly interacts with TGFB1, PLAU, PLAUR, THBS1, FN1, MMP1 (Figure 4).

### 3.5 SERPINE1 Influence on the Immune Microenvironment

**FIGURE 5:**
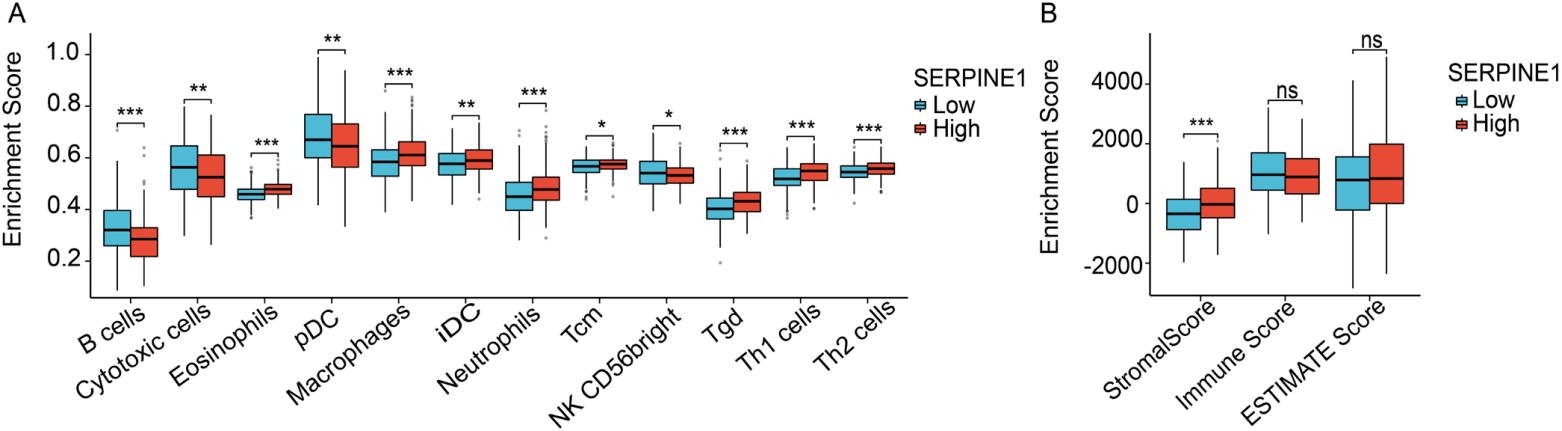
The impact of SERPINE1 on immune cells and the microenvironment. (A) 12 types of immune cells significantly affected by SERPINE1 expression; (B) the relationship between SERPINE1 and immune‐related scores. *p < 0.05, **p < 0.01, ***p < 0.001.

The progression of tumors can be influenced by the infiltration of immune cells. Therefore, we evaluated the correlation between SERPINE1 and the immune microenvironment. We observed a significant positive correlation between SERPINE1 expression and the infiltration levels of eosinophils, Gamma delta T cells (γδ Tcells, Tgd), macrophages, T helper 1 cells (Th1 cells), Th2 cells, Immature dendritic cells (iDC), neutrophils, and Central memory T cells (Tcm cells) (*p* < 0.05). Additionally, there was a significant negative correlation with the levels of cytotoxic cells, B‐cells, Plasmacytoid dendritic cells (pDC), and Natural Killer 56bright cells (NK56bright cells) (*p* < 0.05) (Figure 5A and Table 2). However, there was no significant correlation with the infiltration levels of other cells (Figure S2 and Table S1). Additionally, we observed that tumors with high SERPINE1 expression exhibited significantly higher Stromal Scores, while there was no statistical difference in Immune Score and ESTIMATE Score between the high and low SERPINE1 expression groups (Figure 5B).

**TABLE 2:** Abbreviation list.

Abbreviation	Full name
Eosinophils	Eosinophils
Tgd	Gamma delta T cells (γδ Tcells)
Macrophages	Macrophages
Th1 cells	T helper 1 cells
Th2 cells	T helper 2 cells
iDC	Immature dendritic cells
Neutrophils	Neutrophils
Tcm cells	Central memory T cells
Cytotoxic cells	Cytotoxic cells
B‐cells	B cells
pDC	Plasmacytoid dendritic cells
NK56bright cells	Natural Killer 56bright cells

### 3.6 CNV of SERPINE1 and Methylation Are Associated With the Prognosis of HNSCC

**FIGURE 6:**
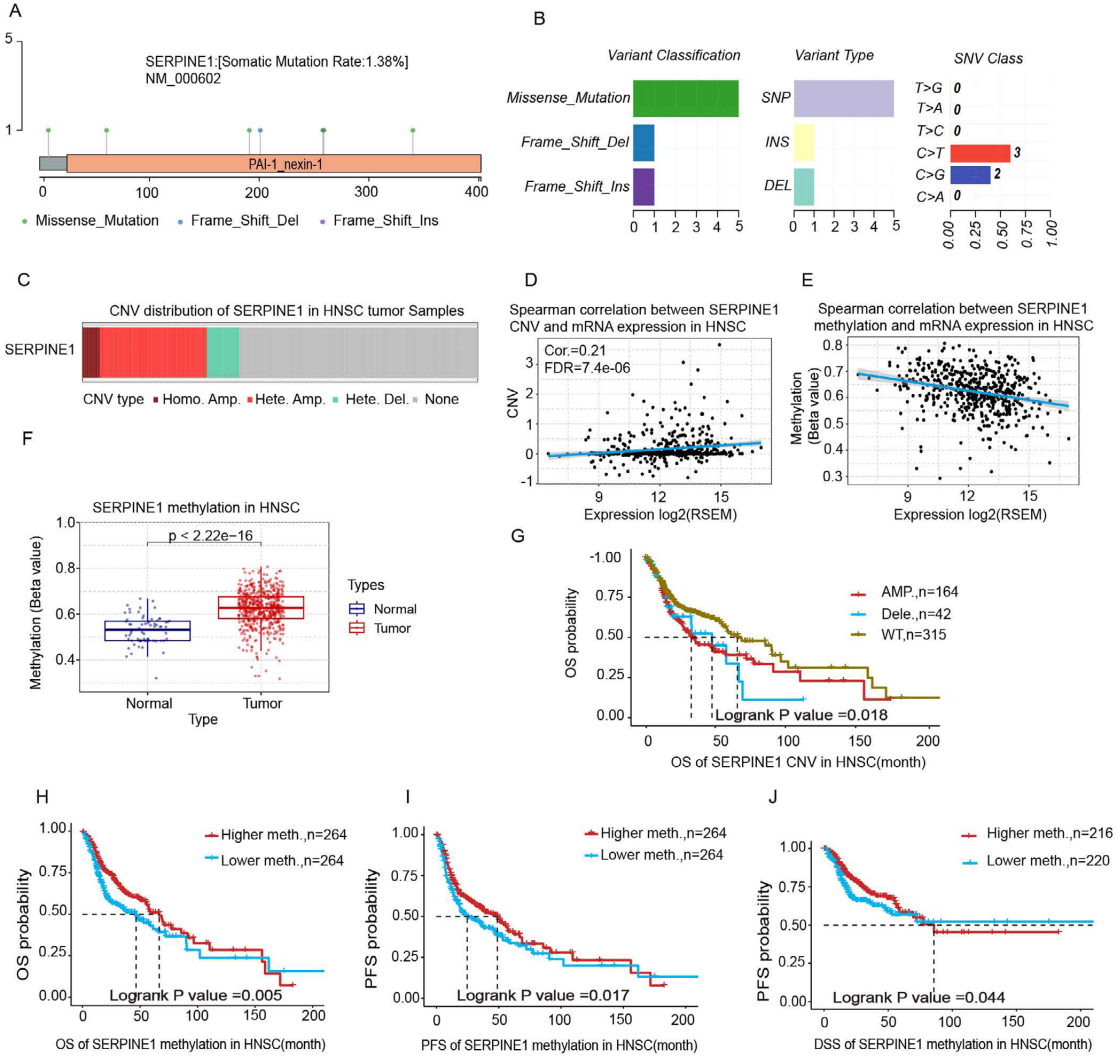
SNV, CNV, and methylation of SERPINE1 and their prognosis. (A, B) SNV of SERPINE1; (C, D) CNV of SERPINE1; (E, F) methylation of SERPINE1; (G–J) the relationship between SERPINE1 expression and survival period.

The SNV profile of SERPINE1 shows a predominance of Missense Mutations, primarily characterized by C > T and C > G mutations (Figure 6A,B). However, these SNVs do not significantly correlate with OS, DSS, or PFS in HNSCC (Figure S3A–C). Analysis of copy number variation (CNV) indicates that SERPINE1 alterations mainly involve Heterogeneous Amplifications (Hete.Amp.), Heterogeneous Deletions (Hete.Del.), and Homogeneous Amplifications (Homo.Amp.) (Figure 6C). CNV of SERPINE1 correlates with its overexpression (Figure 6D). Notably, patients with Wild Type (WT) SERPINE1 exhibit distinct OS, PFS, and DSS outcomes compared to other types, with better prognoses among those with WT SERPINE1 (Figures 6G and S3D,E). Methylation patterns of SERPINE1 are inversely related to its expression, with higher methylation rates observed in HNSCC compared to normal tissues (Figure 6E,F). Significant differences in OS, PFS, and DSS are noted between tumor tissues with high and low methylation rates, with patients having high methylation of SERPINE1 demonstrating improved prognoses (Figure 6H–J).

### 3.7 Drug Sensitivity Assessment

**FIGURE 7:**
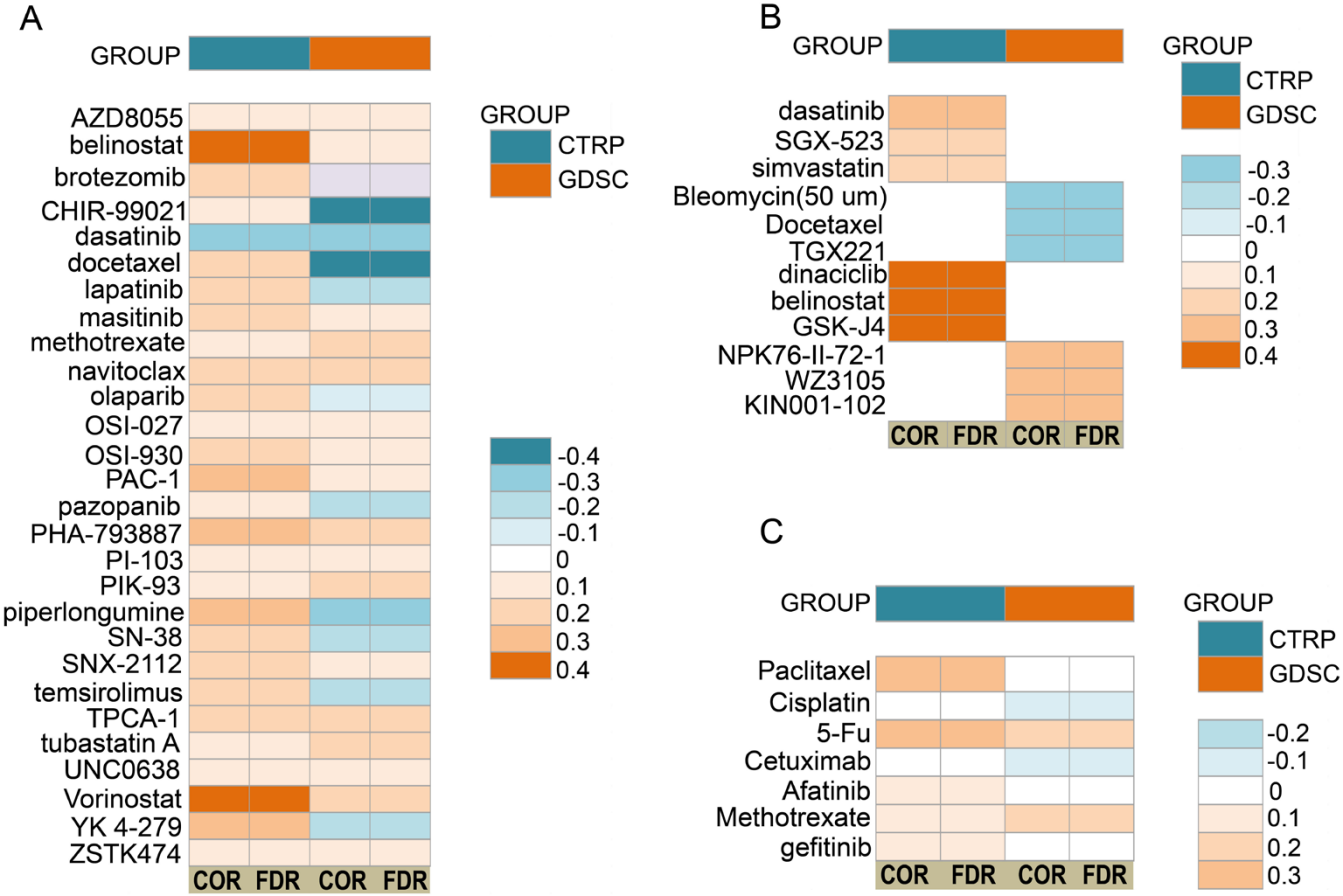
Drugs and SERPINE1 expression in CTRP and GDSC. (A) CTRP and GDSC both show drugs that are associated with SERPINE1; (B) The top three drugs with positive and negative correlation coefficients with SERPINE1 expression in CTRP and GDSC; (C) CTRP and GSDC show the commonly used chemotherapy drugs for HNSCC associated with SERPINE1.

We conducted an analysis of the online GSCA data to identify potential therapeutic drugs. A positive correlation indicates a connection between drug resistance and high gene expression. In Figure 7A, drugs related to SERPINE1 in both CTRP and GDSC databases (FDR < 0.05) are presented. Among them, dasatinib is the only drug showing a negative correlation with SERPINE1 in both databases, while the negative correlation between CHIR99021 and docetaxel with SERPINE1 is more pronounced. Figure 7B displays the top three drugs with positive and negative correlation coefficients with SERPINE1 expression in CTRP and GDSC. Notably, dasatinib, SGX‐523, simvastatin, bleomycin (50 μM), docetaxel, and TGX221 exhibit the smallest negative correlation coefficients with SERPINE1 expression. Figure 7C illustrates the correlation between seven commonly used chemotherapeutic and targeted drugs in the medical treatment of HNSCC. Among them, only cisplatin and cetuximab demonstrate a negative correlation with SERPINE1 expression, while the other drugs exhibit positive correlations.

### 3.8 Immunohistochemistry: High Expression of SERPINE1 in HNSCC

**FIGURE 8:**
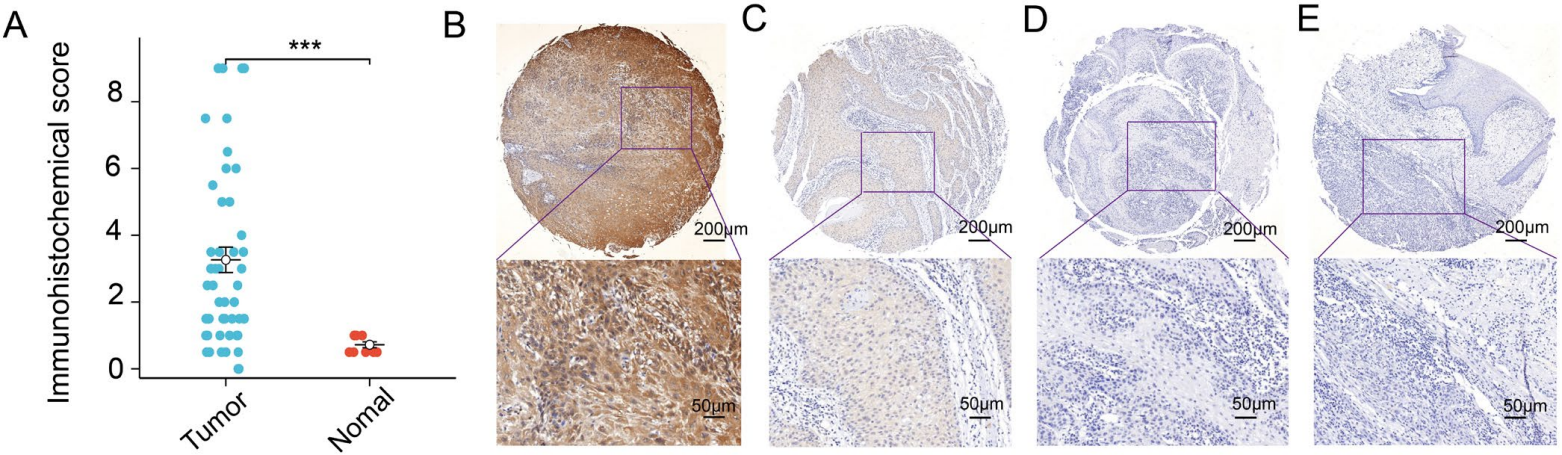
Immunohistochemistry: High expression of SERPINE1 in HNSCC. (A) The immunohistochemical scoring of SERPINE1 in the chip and the correlation between the scoring and clinical indices (*p < 0.05, **p < 0.01, ***p < 0.001); (B) Immunohistochemical staining of SERPINE1 in tumor and adjacent cancer tissues shows strong tumor positivity; (C) positive of tumor; (D) negative of tumor; (E) negative of adjacent tissue (CHIP, the scale above is 200 μm, the scale below is 50 μm).

The tumor cells showed positive staining mainly in the cytoplasm. Comparison of the immunohistochemical scoring of SERPINE1 with the clinical characteristics of patients, as provided by the microarray data, revealed significantly higher immune scoring in patients compared to normal tissue, with statistical significance(Figure 8A). However, no statistical difference was observed between immune scoring and patient gender, age, tumor differentiation, T stage, or lymph node metastasis, consistent with conclusions drawn from TCGA‐HNSCC and GSE65858 datasets (Figure S4). The immunohistochemical study of the chip revealed a statistically significant difference in expression scores between tumor and normal tissues, indicating an upregulation of SERPINE1 expression in HNSCC (Figure 8B–E).

## 4 Discussion

This study investigated the overexpression of SERPINE1 in HNSCC. Our research findings demonstrated a significant upregulation of SERPINE1 in HNSCC, with TCGA data indicating that changes in its expression levels affected OS and DSS. Elevated SERPINE1 expression served as an unfavorable prognostic biomarker, as confirmed by the GSE41613 dataset, which showed significant differences in OS and DSS between patients with high and low SERPINE1 expression. The SE65858 dataset confirmed a significant correlation between SERPINE1 and OS, although there was no statistical difference in the correlation with PFS. It is noteworthy that the predictive value of this gene was confirmed within a 5‐year time frame, and the presence of overlapping OS beyond 5 years may indicate the influence of other external factors on patient survival. The DSS curve revealed no overlap between high and low SERPINE1 expression, indicating a correlation between SERPINE1 and specific tumor‐related survival.

Analysis revealed that SERPINE1, among differentially expressed genes in HNSCC, primarily participates in the AGE‐RAGE signaling pathway in diabetic complications, linked to tumor development [21]. GSEA also highlighted enrichment in pathways related to Extracellular Matrix Organization, ECM Proteoglycans, and others. Additionally, SERPINE1 is implicated in extracellular matrix degradation and resistance to apoptosis during cell migration [22, 23]. The E2F pathway, associated with cell cycle regulation, is often overactivated in cancers, leading to excessive cell proliferation [24]. Furthermore, SERPINE1 interacts with various proteins such as TGFB1, FN1, MMP1, influencing cancer cell adhesion, and motility [25]. TGFB1 notably impacts tumor survival, migration, and metastasis across cancer types [26].

The HNSCC microenvironment is highly immunosuppressive [27]. Bioinformatics analysis revealed positive correlations between SERPINE1 and various immune cells within the tumor microenvironment (TME), including Eosinophils, Tgd, Macrophages, Th1 cells, Th2 cells, iDC, Neutrophils, and Tcm cells, while Cytotoxic cells, B cells, pDC, and NK56 bright cells exhibited negative correlations with SERPINE1. Tumors with high SERPINE1 expression showed elevated Stromal Scores, with no significant differences in Immune Score and ESTIMATE Score between high and low SERPINE1 expression groups. The promotion of specific immune cells within tumors may hinder the anti‐tumor immune response, promoting cancer stem cells and angiogenesis for cancer progression. SERPINE1 may negatively correlate with anti‐tumor immune cells and positively correlate with tumor‐promoting immune cells. Notably, cytotoxic T cells, or CD8+ T cells, directly target tumor cells to induce apoptosis [28]. Macrophage infiltration can either enhance or impede the effectiveness of cancer therapies, while Neutrophils secrete molecules contributing to tumor progression and metastasis [29, 30]. The TME is diverse, with Th2 cells positively correlating with SERPINE1, promoting tumor proliferation and progression [31]. Th1 cells have dual roles, secreting TNF‐α, which, when chronically low, can promote tumor growth, while prolonged exposure can induce immunosuppression [32, 33]. Some studies suggest Th1 cells may prevent tumor progression and induce cell death by activating anti‐tumor cytotoxic and NK cells via IFNγ and IL‐12 in the TME [29, 34].

In this study, we found that SERPINE1 is primarily upregulated in HPV‐negative HNSCC. In patients with HPV‐negative HNSCC who receive postoperative chemoradiation, SERPINE1, HIPDA, CD24, TCF3, INHBA, P4HA2, and ACTN1 are predictors of response [35]. HNSCC exhibits two distinct TME based on HPV infection status. In HPV‐positive HNSCC patients, there is an elevated expression of B cells, plasma cells, Th1 and Th2 CD4+ T cells, CD8+ T cells, regulatory T cells (Tregs), dendritic cells (DCs), and CD56dim NK cells. Conversely, HPV‐negative HNSCC typically exhibits a higher tumor mutation burden (TMB), less infiltration of immune cells, a relatively weaker immune response, and a greater presence of immunosuppressive cells within the TME [36]. The potential influence of SERPINE1 on HPV infection requires further research for confirmation.

Recent discoveries on immune checkpoint inhibitors, such as programmed cell death ligand‐1 (PD‐L1) and programmed cell death‐1 (PD‐1), have led to improved survival rates for various forms of cancer [37]. Two PD‐1 antibodies, Pembrolizumab and Nivolumab, have been approved by the FDA for the treatment of R/M HNSCC after failure of platinum‐based chemotherapy. Notably, SERPINE1 has been significantly correlated with immune checkpoints, particularly PD‐1, PD‐L1, and PD‐L2, and may work synergistically with them [38]. Additionally, SERPINE1 is related to the immune microenvironment. Whether there is a mutual influence between SERPINE1 and PD‐1, PD‐L1, and PD‐L2 warrants further research.

No statistically significant differences were found in SNV of SERPINE1 concerning OS, DSS, and PFS. However, CNV and methylation of SERPINE1 showed significant differences. Specifically, CNV of SERPINE1 did not significantly affect DSS and PFS, but patients with high methylation of SERPINE1 had a better prognosis. Further analysis revealed that Hete.Amp. of CNV might amplify SERPINE1, with its expression negatively correlated with methylation. Patients with low methylation may exhibit high expression of SERPINE1. In summary, CNV and methylation together may contribute to the overexpression of SERPINE1, potentially affecting patient prognosis.

The survival rate for advanced unresectable HNSCC remains low, with first‐line immunotherapy showing an overall response rate (ORR) of 45.9% ± 5.7%, either as monotherapy or in combination with chemotherapy (average ORR 41.5% ± 5.6%) [39]. For patients with disease progression, monotherapy (methotrexate, docetaxel, or cetuximab) typically yields an ORR ranging from 3.9% to 13% [40, 41, 42]. In light of the urgent need for new drugs in HNSCC treatment, our study utilized CTRP and GDSC data from GSCA to explore SERPINE1 and anticancer drugs, aiming to identify potential therapeutic options. The association between SERPINE1 upregulation and chemotherapy suggests a link to chemoresistance, potentially contributing to cisplatin resistance in MIBC [43]. Additionally, EGFR inhibitor therapy induces SERPINE1 expression, correlating with shorter PFS [44]. Our observation of differing therapeutic effects between docetaxel and paclitaxel on SERPINE1 may stem from variances in their mechanisms of action. Despite both drugs belonging to the taxane class, subtle differences in chemical structures and mechanisms may exist. These differences may affect interactions with high SERPINE1‐expressing tumors, thus impacting treatment outcomes. Additionally, tumor heterogeneity, compounded by the use of different tumor cell lines and inherent tumor variability, contributes to this complexity. High SERPINE1‐expressing tumors may exhibit diverse genetic mutations and pathway activations, leading to variable responses to docetaxel and paclitaxel. Thirdly, the emergence of drug resistance is a significant concern, potentially compromising therapeutic efficacy. Even if a tumor initially responds to paclitaxel, it may develop resistance over time, while docetaxel could potentially overcome resistance. However, the intricate relationship between drugs and gene expression necessitates further research.

Due to limited online data availability, we validated SERPINE1 expression in HNSCC using microarray data. Immunohistochemical validation affirmed high SERPINE1 expression in malignant tumors. Despite combining microarray clinical data, no statistical difference was observed in SERPINE1 expression based on patient gender, age, T, and N stage. However, HPV data were lacking in the microarray. Future clinical trials could further validate SERPINE1 expression and its role in HNSCC, while exploring targeted drugs to improve patient outcomes.

## 5 Conclusions

SERPINE1 overexpression in HNSCC suggests its potential as a prognostic biomarker. Its expression levels correlate with the tumor immune microenvironment, supporting the hypothesis that SERPINE1 could serve as a valuable prognostic indicator and a potential therapeutic target.

## Author Contributions

**Changyu Zhu:** data curation (lead), software (lead), writing – original draft (lead). **Heshu Liu:** data curation (supporting). **Zhixin Li:** data curation (supporting). **Yijun Shi:** data curation (supporting). **Jingyang Zhao:** data curation (supporting). **Yuping Bai:** validation (lead). **Qian Chen:** data curation (supporting). **Wei Li:** conceptualization (lead), methodology (lead), writing – review and editing (lead).

## Ethics Statement

In this study, all the data are from public databases and chip data, and the ethical statements of the published public data have been previously published.

## Conflicts of Interest

The authors declare no conflicts of interest.

## Supporting information


Data S1.

## Data Availability

All data and R script in this study are available from the corresponding author upon reasonable request. All authors read and approved the final manuscript. Publicly available datasets were analyzed in this study, and these can be found in The Cancer Genome Atlas (https://portal.gdc.cancer.gov/) and Gene Expression (GSE65858 and GSE41613).

## References

[1] J. Ferlay , I. Soerjomataram , R. Dikshit , et al., “Cancer Incidence and Mortality Worldwide: Sources, Methods and Major Patterns in GLOBOCAN 2012,” International Journal of Cancer 136, no. 5 (2015): E359–E386. 25220842 10.1002/ijc.29210

[2] C. R. Leemans , B. J. Braakhuis , and R. H. Brakenhoff , “The Molecular Biology of Head and Neck Cancer,” Nature Reviews Cancer 11, no. 1 (2011): 9–22. 21160525 10.1038/nrc2982

[3] A. E. Zou , H. Zheng , M. A. Saad , et al., “The Non‐coding Landscape of Head and Neck Squamous Cell Carcinoma,” Oncotarget 7, no. 32 (2016): 51211–51222. 27323410 10.18632/oncotarget.9979 PMC5239470

[4] T. L. Whiteside , “Head and Neck Carcinoma Immunotherapy: Facts and Hopes,” Clinical Cancer Research 24, no. 1 (2018): 6–13. 28751445 10.1158/1078-0432.CCR-17-1261 PMC5754223

[5] Z. Mei , K. Zhang , A. K. Y. Lam , et al., “MUC1 as a Target for CAR‐T Therapy in Head and Neck Squamous Cell Carinoma,” Cancer Medicine 9, no. 2 (2020): 640–652. 31800160 10.1002/cam4.2733 PMC6970025

[6] C. Dellas and D. J. Loskutoff , “Historical Analysis of PAI‐1 From Its Discovery to Its Potential Role in Cell Motility and Disease,” Thrombosis and Haemostasis 93, no. 4 (2005): 631–640. 15841306 10.1160/TH05-01-0033

[7] M. H. Kubala and Y. A. DeClerck , “The Plasminogen Activator Inhibitor‐1 Paradox in Cancer: A Mechanistic Understanding,” Cancer and Metastasis Reviews 38, no. 3 (2019): 483–492. 31734763 10.1007/s10555-019-09806-4 PMC7001780

[8] G. Mazzoccoli , V. Pazienza , A. Panza , et al., “ARNTL2 and SERPINE1: Potential Biomarkers for Tumor Aggressiveness in Colorectal Cancer,” Journal of Cancer Research and Clinical Oncology 138 (2012): 501–511. 22198637 10.1007/s00432-011-1126-6 PMC11824804

[9] E. Jakubowicz , B. Martin , R. Hoffmann , et al., “EndoPredict Versus uPA/PAI‐1 in Breast Cancer: Comparison of Markers and Association With Clinicopathological Parameters,” Breast Journal 25, no. 3 (2019): 450–454. 31001905 10.1111/tbj.13258

[10] S. M. Akula , P. P. Ruvolo , and J. A. McCubrey , “TP53/miR‐34a‐Associated Signaling Targets SERPINE1 Expression in Human Pancreatic Cancer,” Aging (Albany NY) 12, no. 3 (2020): 2777–2797. 31986125 10.18632/aging.102776 PMC7041729

[11] H. Furuya , Y. Sasaki , R. Chen , et al., “PAI‐1 Is a Potential Transcriptional Silencer That Supports Bladder Cancer Cell Activity,” Scientific Reports 12, no. 1 (2022): 12186. 35842542 10.1038/s41598-022-16518-3 PMC9288475

[12] H. Yang , Z. Wang , L. Gong , et al., “Yang X (2022) A Novel Hypoxia‐Related Gene Signature With Strong Predicting Ability in Non‐Small‐Cell Lung Cancer Identified by Comprehensive Profiling,” International Journal of Genomics 2022 (2022): 1–18. 10.1155/2022/8594658 PMC9135579 35634481

[13] F. Seker , A. Cingoz , I. Sur‐Erdem , et al., “Identification of SERPINE1 as a Regulator of Glioblastoma Cell Dispersal With Transcriptome Profiling,” Cancers 11, no. 11 (2019): 1651. 31731490 10.3390/cancers11111651 PMC6896086

[14] Y. Wang , J. Wang , J. Gao , M. Ding , and H. Li , “The Expression of SERPINE1 in Colon Cancer and Its Regulatory Network and Prognostic Value,” BMC Gastroenterology 23, no. 1 (2023): 33. 36755247 10.1186/s12876-022-02625-y PMC9906885

[15] J.‐D. Yang , L. Ma , and Z. Zhu , “SERPINE1 as a Cancer‐Promoting Gene in Gastric Adenocarcinoma: Facilitates Tumour Cell Proliferation, Migration, and Invasion by Regulating EMT,” Journal of Chemotherapy 31, no. 7–8 (2019): 408–418. 31724495 10.1080/1120009X.2019.1687996

[16] F. Teng , J. X. Zhang , Y. Chen , et al., “LncRNA NKX2‐1‐AS1 Promotes Tumor Progression and Angiogenesis via Upregulation of SERPINE1 Expression and Activation of the VEGFR‐2 Signaling Pathway in Gastric Cancer,” Molecular Oncology 15, no. 4 (2021): 1234–1255. 33512745 10.1002/1878-0261.12911 PMC8024734

[17] M. A. Pavón , I. Arroyo‐Solera , M. V. Céspedes , I. Casanova , X. León , and R. Mangues , “uPA/uPAR and SERPINE1 in Head and Neck Cancer: Role in Tumor Resistance, Metastasis, Prognosis and Therapy,” Oncotarget 7, no. 35 (2016): 57351–57366. 27385000 10.18632/oncotarget.10344 PMC5302994

[18] G. Yu , L.‐G. Wang , Y. Han , and Q.‐Y. He , “clusterProfiler: An R Package for Comparing Biological Themes Among Gene Clusters,” OMICS: A Journal of Integrative Biology 16, no. 5 (2012): 284–287. 22455463 10.1089/omi.2011.0118 PMC3339379

[19] G. Bindea , B. Mlecnik , M. Tosolini , et al., “Spatiotemporal Dynamics of Intratumoral Immune Cells Reveal the Immune Landscape in Human Cancer,” Immunity 39, no. 4 (2013): 782–795. 24138885 10.1016/j.immuni.2013.10.003

[20] C.‐J. Liu , F.‐F. Hu , G.‐Y. Xie , et al., “GSCA: An Integrated Platform for Gene Set Cancer Analysis at Genomic, Pharmacogenomic and Immunogenomic Levels,” Briefings in Bioinformatics 24, no. 1 (2023): bbac558. 36549921 10.1093/bib/bbac558

[21] B. Yu , G. Lv , M. Sohail , et al., “Utilizing Bioinformatics Technology to Explore the Potential Mechanism of Danggui Buxue Decoction Against NSCLC,” Disease Markers 2022 (2022): 1–20. 10.1155/2022/5296830 PMC8898125 35256890

[22] E. G. Giacoia , M. Miyake , A. Lawton , S. Goodison , and C. J. Rosser , “PAI‐1 Leads to G1‐Phase Cell‐Cycle Progression Through Cyclin D3/cdk4/6 Upregulation,” Molecular Cancer Research 12, no. 3 (2014): 322–334. 24464915 10.1158/1541-7786.MCR-13-0543 PMC4064567

[23] F. Seker , A. Cingoz , İ. Sur‐Erdem , et al., “Identification of SERPINE1 as a Regulator of Glioblastoma Cell Dispersal With Transcriptome Profiling,” Cancers (Basel) 11, no. 11 (2019): 1651. 31731490 10.3390/cancers11111651 PMC6896086

[24] Y. Du , W. Jiang , S. Hou , Z. Chen , and W. Zhou , “A Novel Cuproptosis‐Associated Gene Signature to Predict Prognosis in Patients With Pancreatic Cancer,” BioMed Research International 2023 (2023): 1–15. 10.1155/2023/3419401 PMC9876676 36714025

[25] R. Wang , X. Zhou , H. Wang , et al., “Integrative Analysis of Gene Expression Profiles Reveals Distinct Molecular Characteristics in Oral Tongue Squamous Cell Carcinoma,” Oncology Letters 17, no. 2 (2019): 2377–2387. 30675303 10.3892/ol.2018.9866 PMC6341834

[26] B. Kaminska , A. Wesolowska , and M. Danilkiewicz , “TGF Beta Signalling and Its Role in Tumour Pathogenesis,” Acta Biochimica Polonica 52, no. 2 (2005): 329–337. 15990918

[27] M.‐N. Theodoraki , S. S. Yerneni , T. K. Hoffmann , W. E. Gooding , and T. L. Whiteside , “Clinical Significance of PD‐L1+ Exosomes in Plasma of Head and Neck Cancer Patients,” Clinical Cancer Research 24, no. 4 (2018): 896–905. 29233903 10.1158/1078-0432.CCR-17-2664 PMC6126905

[28] L. Chen , Y. Li , and X. Deng , “Comprehensive Analysis of Pan‐Cancer Reveals the Potential of SLC16A1 as a Prognostic and Immunological Biomarker,” Medicine 102, no. 11 (2023): e33242. 36930112 10.1097/MD.0000000000033242 PMC10019278

[29] N. Nagarsheth , M. S. Wicha , and W. Zou , “Chemokines in the Cancer Microenvironment and Their Relevance in Cancer Immunotherapy,” Nature Reviews Immunology 17, no. 9 (2017): 559–572. 10.1038/nri.2017.49 PMC5731833 28555670

[30] J. E. De Larco , B. R. Wuertz , and L. T. Furcht , “The Potential Role of Neutrophils in Promoting the Metastatic Phenotype of Tumors Releasing Interleukin‐8,” Clinical Cancer Research 10, no. 15 (2004): 4895–4900. 15297389 10.1158/1078-0432.CCR-03-0760

[31] E. Osawa , A. Nakajima , T. Fujisawa , et al., “Predominant T Helper Type 2‐Inflammatory Responses Promote Murine Colon Cancers,” International Journal of Cancer 118, no. 9 (2006): 2232–2236. 16331625 10.1002/ijc.21639

[32] F. Balkwill , “TNF‐α in Promotion and Progression of Cancer,” Cancer and Metastasis Reviews 25 (2006): 409–416. 16951987 10.1007/s10555-006-9005-3

[33] X. Chen , M. Bäumel , D. N. Männel , O. Howard , and J. J. Oppenheim , “Interaction of TNF With TNF Receptor Type 2 Promotes Expansion and Function of Mouse CD4+ CD25+ T Regulatory Cells,” Journal of Immunology 179, no. 1 (2007): 154–161. 10.4049/jimmunol.179.1.154 17579033

[34] D. Compare and G. Nardone , “Contribution of Gut Microbiota to Colonic and Extracolonic Cancer Development,” Digestive Diseases 29, no. 6 (2011): 554–561. 22179211 10.1159/000332967

[35] S. Schmidt , A. Linge , A. Zwanenburg , et al., “Development and Validation of a Gene Signature for Patients With Head and Neck Carcinomas Treated by Postoperative Radio(Chemo)therapy,” Clinical Cancer Research 24, no. 6 (2018): 1364–1374, 10.1158/1078-0432.CCR-17-2345. 29298797

[36] K. Starska‐Kowarska , “The Role of Different Immunocompetent Cell Populations in the Pathogenesis of Head and Neck Cancer‐Regulatory Mechanisms of Pro‐ and Anti‐Cancer Activity and Their Impact on Immunotherapy,” Cancers (Basel) 15, no. 6 (2023): 1642, 10.3390/cancers15061642. 36980527 PMC10046400

[37] X. Dong , J. Fan , W. Xie , et al., “Efficacy Evaluation of Chimeric Antigen Receptor‐Modified Human Peritoneal Macrophages in the Treatment of Gastric Cancer,” British Journal of Cancer 129, no. 3 (2023): 551–562, 10.1038/s41416-023-02319-6. 37386139 PMC10403530

[38] X. Huang , F. Zhang , D. He , et al., “Immune‐Related Gene SERPINE1 Is a Novel Biomarker for Diffuse Lower‐Grade Gliomas via Large‐Scale Analysis,” Frontiers in Oncology 20, no. 11 (2021): 646060, 10.3389/fonc.2021.646060. PMC8173178 34094933

[39] N. Amin , C. A. Maroun , M. El Asmar , et al., “Neoadjuvant Immunotherapy Prior to Surgery for Mucosal Head and Neck Squamous Cell Carcinoma: Systematic Review,” Head & Neck 44, no. 2 (2022): 562–571. 34825751 10.1002/hed.26935

[40] J. S. W. Stewart , E. E. Cohen , L. Licitra , et al., “Phase III Study of Gefitinib Compared With Intravenous Methotrexate for Recurrent Squamous Cell Carcinoma of the Head and Neck,” Journal of Clinical Oncology 27, no. 11 (2009): 1864–1871. 19289630 10.1200/JCO.2008.17.0530

[41] J. B. Vermorken , J. Trigo , R. Hitt , et al., “Open‐Label, Uncontrolled, Multicenter Phase II Study to Evaluate the Efficacy and Toxicity of Cetuximab as a Single Agent in Patients With Recurrent and/or Metastatic Squamous Cell Carcinoma of the Head and Neck Who Failed to Respond to Platinum‐Based Therapy,” Journal of Clinical Oncology 25, no. 16 (2007): 2171–2177. 17538161 10.1200/JCO.2006.06.7447

[42] S. Zenda , Y. Onozawa , N. Boku , Y. Iida , M. Ebihara , and T. Onitsuka , “Single‐Agent Docetaxel in Patients With Platinum‐Refractory Metastatic or Recurrent Squamous Cell Carcinoma of the Head and Neck (SCCHN),” Japanese Journal of Clinical Oncology 37, no. 7 (2007): 477–481. 17720737 10.1093/jjco/hym059

[43] R. M. Gil da Costa , C. Levesque , D. Bianchi‐Frias , et al., “Pharmacological NF‐κB Inhibition Decreases Cisplatin Chemoresistance in Muscle‐Invasive Bladder Cancer and Reduces Cisplatin‐Induced Toxicities,” Molecular Oncology 17, no. 12 (2023): 2709–2727. 37533407 10.1002/1878-0261.13504 PMC10701775

[44] R. R. Arasada , K. Shilo , T. Yamada , et al., “Notch3‐Dependent β‐Catenin Signaling Mediates EGFR TKI Drug Persistence in EGFR Mutant NSCLC,” Nature Communications 9, no. 1 (2018): 3198. 10.1038/s41467-018-05626-2 PMC6090531 30097569

