# Supplementary material for: Prognostic Significance and Therapeutic Potential of SERPINE1 in Head and Neck Squamous Cell Carcinoma
Source: Cancer Med. 2025 Jan 16;14(2):e70605. doi: 10.1002/cam4.70605 (PMC11736624; doi:10.1002/cam4.70605)
Supplement: Supplementary file 1 — Data S1. [file CAM4-14-e70605-s001.docx]

Supplementary Table1: Abbreviation list

| **Abbreviation** | **Full name** |
| --- | --- |
| NK CD56dim cells | Natural Killer CD56dim cells |
| aDC | Activated dendritic cells |
| Tem | Effector memory T cells |
| mAST CELLS | Mast cells |
| TFH | T follicular helper cells |
| th17 cells | T helper 17 cells |
| TReg | Regulatory T cells |


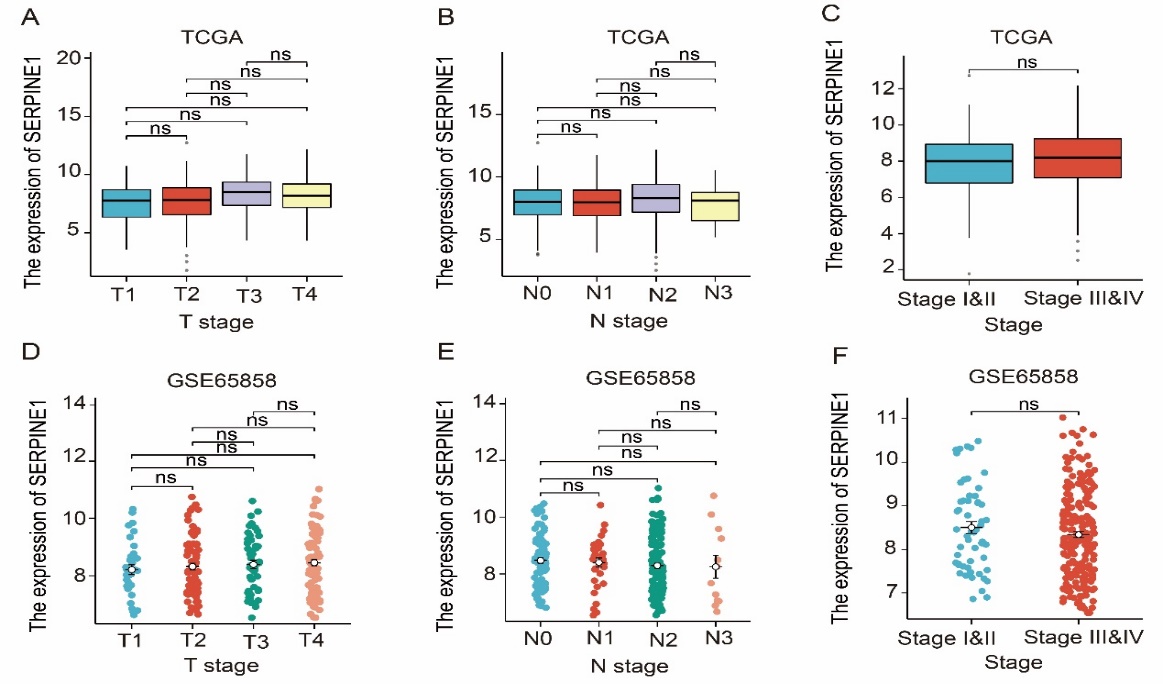


Supplementary Figure 1 The association between SERPINE1 and clinical features

A-C, The relationship between SERPINE1 expression and T stage, N stage and pathological stage(TCGA).

D-F, The relationship between SERPINE1 expression and T stage, N stage and pathological stage(GSE65858).


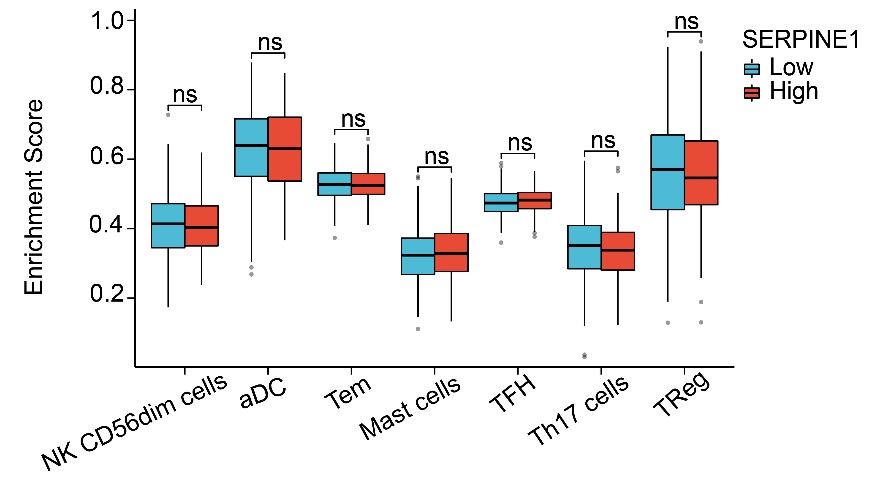


Supplementary Figure 2 7 Types of Immune Cells Not Significantly Affected by SERPINE1 Expression


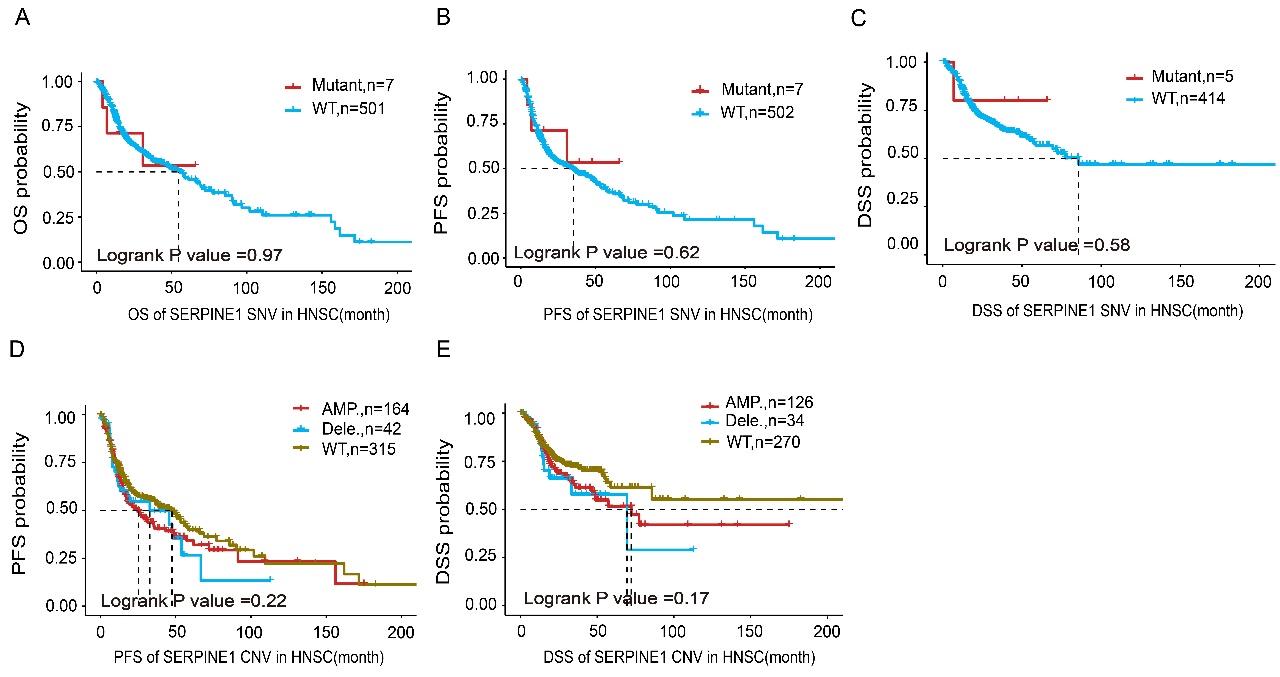


Supplementary Figure 3 The relationship between SERPINE1 expression and survival period.


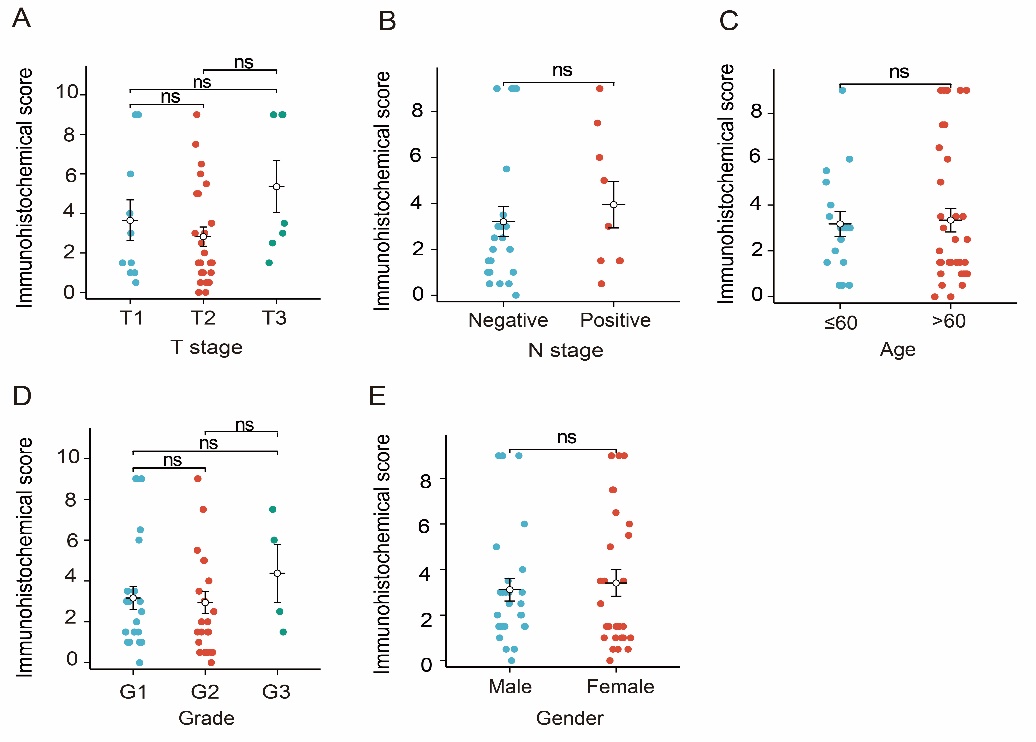


Supplementary Figure 4 The immunohistochemical scoring of SERPINE1 in the chip and the correlation between the scoring and clinical indices
